# Statistical analysis of genomic protein family and domain controlled annotations for functional investigation of classified gene lists

**DOI:** 10.1186/1471-2105-8-S1-S14

**Published:** 2007-03-08

**Authors:** Marco Masseroli, Elisa Bellistri, Andrea Franceschini, Francesco Pinciroli

**Affiliations:** 1 Dipartimento di Elettronica e Informazione, Politecnico di Milano, piazza Leonardo da Vinci 32, 20133 Milano, Italy; 2 BioMedical Informatics Laboratory, Dipartimento di Bioingegneria, Politecnico di Milano, piazza Leonardo da Vinci 32, 20133 Milano, Italy

## Abstract

**Background:**

The increasing protein family and domain based annotations constitute important information to understand protein functions and gain insight into relations among their codifying genes. To allow analyzing of gene proteomic annotations, we implemented novel modules within *GFINDer*, a Web system we previously developed that dynamically aggregates functional and phenotypic annotations of user-uploaded gene lists and allows performing their statistical analysis and mining.

**Results:**

Exploiting protein information in Pfam and InterPro databanks, we developed and added in *GFINDer *original modules specifically devoted to the exploration and analysis of functional signatures of gene protein products. They allow annotating numerous user-classified nucleotide sequence identifiers with controlled information on related protein families, domains and functional sites, classifying them according to such protein annotation categories, and statistically analyzing the obtained classifications. In particular, when uploaded nucleotide sequence identifiers are subdivided in classes, the *Statistics Protein Families&Domains *module allows estimating relevance of Pfam or InterPro controlled annotations for the uploaded genes by highlighting protein signatures significantly more represented within user-defined classes of genes. In addition, the *Logistic Regression *module allows identifying protein functional signatures that better explain the considered gene classification.

**Conclusion:**

Novel *GFINDer *modules provide genomic protein family and domain analyses supporting better functional interpretation of gene classes, for instance defined through statistical and clustering analyses of gene expression results from microarray experiments. They can hence help understanding fundamental biological processes and complex cellular mechanisms influenced by protein domain composition, and contribute to unveil new biomedical knowledge about the codifying genes.

## Background

A great quantity of biomolecular information about genes and proteins is increasingly accumulating in form of textual annotations within heterogeneous and widely distributed databanks [1], which are often publicly accessible through the Internet. Among such information, gene function controlled annotations are the most interesting because their analysis can highlight new biomedical knowledge, including the identification of functional relationships among genes or the involvement of specific genes in complex patho-physiological processes.

The gene Ontology (GO) [2] is currently considered the most important source of functional information and numerous studies are based on GO biological processes or molecular function controlled annotations. However, in many cases the Gene Ontology provides only high level annotations of processes or functions that can be associated with many disorders and in which numerous genes can be involved. Moreover, many genes and proteins still have very few or no GO annotations, and several GO annotations have been assigned only according to computational inferences but not yet experimentally confirmed or revised by experts. Thus, to investigate gene function at a genomic level we think it is important to consider all different types of functional information available. Among them protein family and domain annotations constitute one of the most useful information to understand protein functions and gain insight into relations among their codifying genes [3]. Comprehension of domain structure of proteins within genomes is also fundamental to better understand the evolutionary forces and emerging functions shaping genomes [3]. In fact, protein families and domains respectively represent groups of evolutionarily related proteins and their components that fold independently from the remaining protein chain. The increasing number of proteins for which domain-based annotations are available hence represents an important background for computational genome-wise analyses [3].

In the last years, several databanks have been creating to collect and provide proteomic information in organized form, including protein family and domain controlled annotations. Among them, the Pfam [4] and InterPro [5] databanks are considered the most reliable and comprehensive, respectively. Pfam is a collection of protein families and domains that contains information about multiple alignments of protein domains and conserved protein regions. InterPro is an integrated documentation resource for protein families, domains and functional sites. It combines a number of databases, which use different methodologies and a varying degree of biological information on well-characterized proteins to derive protein signatures. Pfam, InterPro and other databanks supply a great amount of valuable information. However, for the interpretation of large experimental datasets, such as those from high-throughput technologies, it is paramount the support of automated tools able to automatically retrieve the information of interest from these databanks and use it for exhaustive analysis and mining.

In order to allow comprehensive evaluation of gene annotations sparsely available in numerous different databanks accessible through the Internet, we previously developed *GFINDer *[6]. It is a publicly accessible Web server [7] that dynamically aggregates and keeps updated functional and phenotypic annotations of user-uploaded gene lists and allows performing their statistical analysis and mining. For this purpose, in *GFINDer *independent and interconnected modules use several controlled vocabularies describing gene related biomolecular processes and functions.

With the aim of effectively take advantage of the valuable protein information present in Pfam and InterPro databanks, in *GFINDer *we implemented new specific modules dedicated to exploring and analyzing functional signatures of gene protein products. They allow annotating user-classified nucleotide sequence identifiers with controlled information on codified protein families, domains, and functional sites; classifying them according to such protein annotation categories; and statistically analyzing the classifications defined. The novel *GFINDer *modules support a genomic approach in the biological interpretation of high-throughput experimental results and hence in the understanding of fundamental biological processes and complex cellular patho-physiological mechanisms.

## Results

### Structuring and hierarchical tree reconstruction of protein family and domain controlled annotations

Pfam databank version 19.0 contained 8,183 protein family domain entries and InterPro databank release 12.1 included 12,967 entries (9,065 protein families, 3,587 protein domains and 315 functional sites including post translational modifications, repeats, active and binding sites). We structured all these entries in a *GFINDer *database and found that 3,495 InterPro entries (2,587 protein families, 877 protein domains and 31 functional sites) were grouped in 927 hierarchical trees of parent/child relations (610 of protein families, 304 of protein domains and 13 of functional sites). We reconstructed such parent/child hierarchical trees within the *GFINDer *database and noticed that protein family trees had a maximum of 6 levels, with an average of 431 entries per level; protein domain trees had a maximum of 5 levels, with an average of 175 entries per level; and functional site trees had a maximum of 2 levels, with an average of 14 entries per level. Thus, only a total of 26.95% of the protein family (28.54%), domain (24.45%) and functional sites (9.84%) controlled annotations available in InterPro are hierarchically organized in many unrelated and very large, but not deep, trees of parent/child hierarchical relations.

### Statistical analysis of protein family and domain annotations

**Figure 1:**
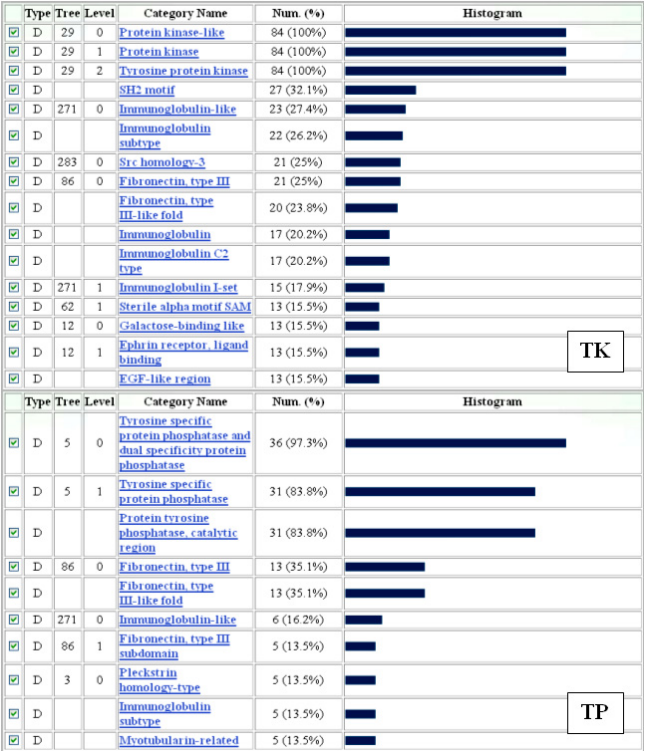
***GFINDer Exploration Protein Familes&Domains *module: protein domain exploration**. InterPro protein domains related to the considered tyrosine kinase (TK) or tyrosine phosphatase (TP) gene classes; Type: type of InterPro entry (D: protein domain); Tree: tree label in the defined protein domain parent/child hierarchy, if any; Level: level in the related tree of the defined protein domain parent/child hierarchy, if any (higher levels correspond to more specific protein domains); Num. (%): absolute and percentage number of considered genes that codify proteins containing the specific protein domain (Category Name).

**Figure 2:**
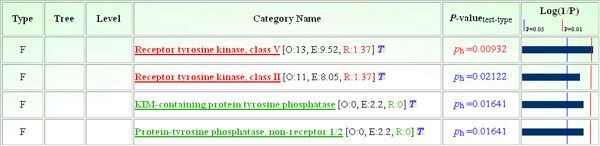
***GFINDer Statistics Protein Familes&Domains *module: protein family analysis**. InterPro protein families most significantly over- (red) and under-represented (green) in the considered tyrosine kinase versus tyrosine phosphatase gene classes; Type: type of InterPro entry (F: protein family); Tree: tree label in the defined protein family parent/child hierarchy, if any; Level: level in the related tree of the defined protein family parent/child hierarchy, if any (higher levels correspond to more specific protein families); *P*-value_test-type_: *p *value defining relevance of a given protein family (Category Name) for a considered class of genes, and initial of used statistical test name (h: hypergeometric distribution test).

**Figure 3:**
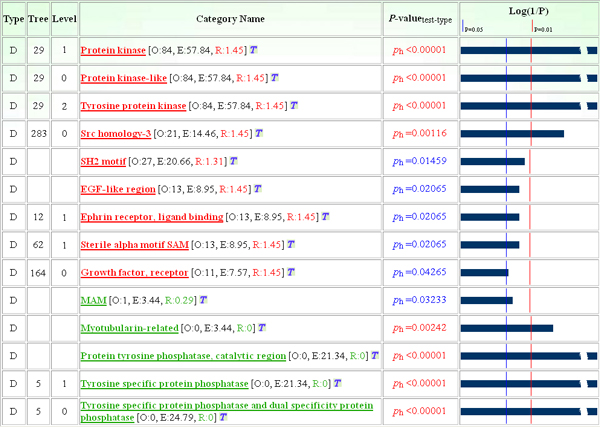
***GFINDer Statistics Protein Familes&Domains *module: protein domain analysis**. InterPro protein domains most significantly over- (red) and under-represented (green) in the considered tyrosine kinase versus tyrosine phosphatase gene classes; Type: type of InterPro entry (D: protein domain); Tree: tree label in the defined protein domain parent/child hierarchy, if any; Level: level in the related tree of the defined protein domain parent/child hierarchy, if any (higher levels correspond to more specific protein domains); *P*-value_test-type_: *p *value defining relevance of a given protein domain (Category Name) for a considered class of genes, and initial of used statistical test name (h: hypergeometric distribution test).

We developed and added within the *GFINDer *Web system new original modules specifically devoted to the exploration and analysis of functional signatures of gene protein products. They first enable the annotation of numerous user-classified nucleotide sequence identifiers with controlled information on related protein families, domains, and functional sites available in Pfam and InterPro databanks. Then, they allow classifying the annotated nucleotide sequence identifiers according to their protein annotation categories, and statistically analyzing the obtained classifications. In particular, the *Exploration Protein Families&Domains *module (Figure 1) allows to easily and graphically understand either how many and which protein families, domains, and functional sites are associated with each considered gene, or how many of the user-selected genes refer to each protein family, domain, or functional site. When the user-uploaded genes are subdivided in classes (e.g. from clustering analysis of microarray results), the *Statistics Protein Families&Domains *module (Figures 2 and 3) allows evaluating the importance of each Pfam or InterPro controlled annotation for the uploaded genes by highlighting protein annotations significantly more represented within the user-defined classes of genes. The annotated genes are grouped accordingly to their class and annotation categories, and their distribution among the considered categories is statistically evaluated. For this aim, different statistical tests and type of corrections for multiple tests have been implemented in *GFINDer *(see the Methods section). After selecting a specific gene class, for each protein family or domain annotation category in that class the module provides the observed number of input genes, their expected number, and the significance *p*-value for that category with its histogram (Figures 2 and 3). In addition, external links to Pfam or InterPro protein family or domain descriptions related to the considered genes are given.

### Logistic regression of protein family and domain annotations

In *GFINDer *Web system we also implemented a new *Logistic Regression *module. It exploits controlled protein family and domain information provided by Pfam and InterPro databanks to allow executing logistic regression analysis of functional signature annotations that characterize protein products of user-uploaded classified gene lists. Such analysis can allow identifying which protein functional characteristics better explain the binary classification of a set of genes, for instance obtained through statistical and clustering analysis of gene expression results from microarray experiments. It can also allow estimating the probability that a gene, which codifies proteins with certain functional characteristics, is functionally similar to a set of genes whose protein products have those functional characteristics. This can help in revealing or better understanding the functions of new or less studied genes.

**Figure 4:**
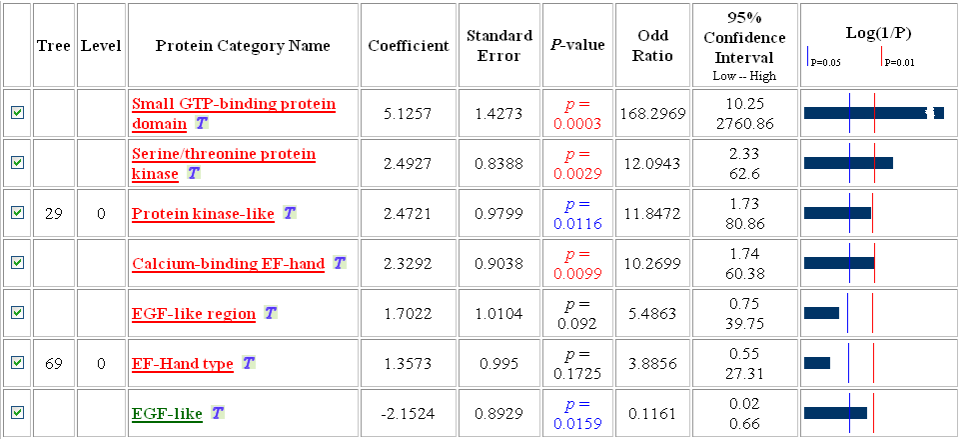
***GFINDer Logistic Regression *module: selected protein domains**. InterPro protein domains (Protein Category Name) with a relevant logistic regression coefficient and codified by the considered neurobiology or cardiovascular system related genes; Tree: tree label in the defined protein domain parent/child hierarchy, if any; Level: level in the related tree of the defined protein domain parent/child hierarchy, if any (higher levels correspond to more specific protein domains).

**Figure 5:**
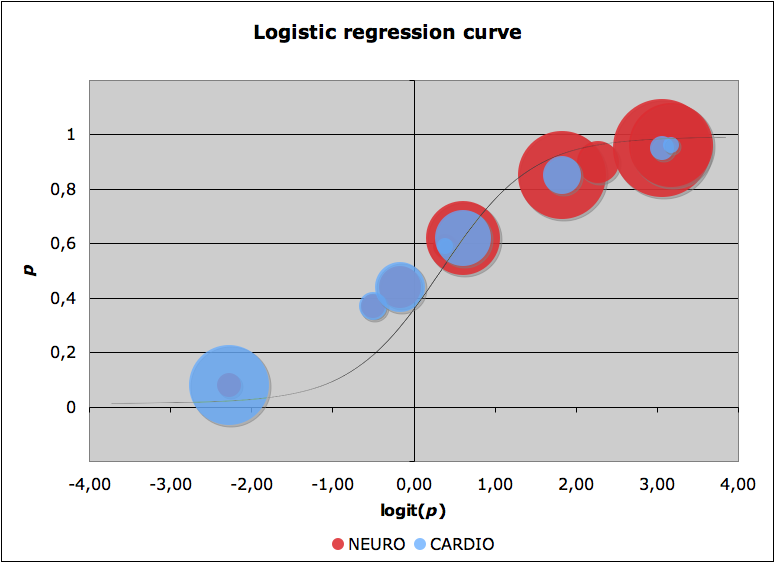
**Calculated logistic regression curve for the considered neurobiology (NEURO) or cardiovascular system (CARDIO) related genes**. Each point area indicates the number of considered genes with a calculated regression value (logit(*p*)) and estimated probability (*p*), of belonging to the NEURO class, corresponding to the point center.

Figure 4 shows an example of logistic regression analysis results obtained for a set of genes whose protein product domains and relation to neurobiology (121 genes), or cardiovascular system (55 genes), are known [8-10]. Figure 5 shows the distribution of such considered genes according to their values of calculated logistic regression and estimated probability of being related to neurobiology.

### Validation of implemented protein family and domain analysis

In order to test effectiveness of our approach and assess capabilities of implemented *GFINDer Protein Familes&Domains *modules, we used them to evaluate two distinct sets of genes. The first consisted of 142 genes codifying tyrosine kinase proteins (90 genes) or tyrosine phosphatase proteins (52 genes) [11-13]. The second included 179 genes related to apoptosis (92 genes) or growth factors (87 genes) [14-16]. We used the *GFINDer Exploration *and *Statistics Protein Families&Domains *modules to evaluate the relevant presence of genes associated with specific protein families or domains within the tyrosine kinase genes (TK) versus the tyrosine phosphatase genes (TP), or within the apoptosis genes (APO) versus the growth factor genes (GF) considered.

First, with the *Exploration *module we observed the distribution of protein families or domains within the two considered TK and TP classes of genes (Figure 1). Then, using the *Statistics *module we evaluated the protein families most represented in the TK versus TP class. We concentrated only on genes with protein family annotations and on protein family categories associated with at least three of the considered genes. As shown in Figure 2, statistical analysis correctly selected protein families related to the appropriate class of considered genes. In fact, the significant protein families selected included: "Receptor tyrosine kinase, class V" (*p *= 0.00932) and "Receptor tyrosine kinase, class II" (*p *= 0.02122) categories for the TK class; "Protein-tyrosine phosphatase, non-receptor 1/2" (*p *= 0.01641) and "KIM-containing protein tyrosine phosphatase" (*p *= 0.01641) categories for the TP class.

Finally, we analyzed the protein domains most represented in the TK versus TP class. Focusing only on genes with protein domain annotations and on protein domain categories associated with at least five of the considered genes, *GFINDer *statistical analysis properly highlighted as most relevant in each gene class protein domains logically pertaining to that class (Figure 3). In fact, the protein domains selected as most significant after FDR multiple test correction included: "Protein kinase" (*p *< 0.00001), "Protein kinase-like" (*p *< 0.00001), "Tyrosine protein kinase" (*p *< 0.00001), "Src homology-3" (*p *= 0.00016), and "SH2 motif" (*p *= 0.01459) protein domains for the TK gene class; "Tyrosine specific protein phosphatase and dual specificity protein phosphatase" (*p *< 0.00001), "Tyrosine specific protein phosphatase" (*p *< 0.00001), and "Protein tyrosine phosphatase, catalytic region" (*p *< 0.00001) for the TP class.

**Figure 6:**
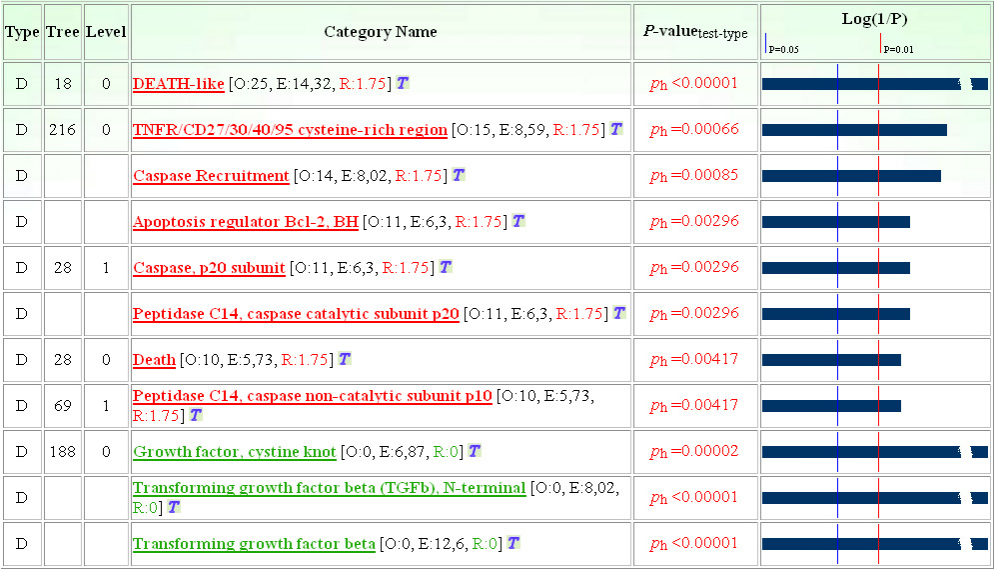
***GFINDer Statistics Protein Familes&Domains *module: protein domain analysis**. InterPro protein domains most significantly over- (red) and under-represented (green) in the considered apoptosis versus growth factor related gene classes; Type: type of InterPro entry (D: protein domain); Tree: tree label in the defined protein domain parent/child hierarchy, if any; Level: level in the related tree of the defined protein domain parent/child hierarchy, if any (higher levels correspond to more specific protein domains); *P*-value_test-type_: *p *value defining relevance of a given protein domain (Category Name) for a considered class of genes, and initial of used statistical test name (h: hypergeometric distribution test).

Similarly, we used the *Statistics *module to evaluate the protein domains most represented in the APO versus GF class. Also in this case, *GFINDer *statistical analysis properly selected as most relevant in each gene class protein domains correctly pertaining to that class (Figure 6). In fact, they included "DEATH-like" (*p *< 0.00001), "TNFR/CD27/30/40/95 cysteine-rich region" (*p *= 0.00066), "Caspase Recruitment" (*p *= 0.00085), and "Apoptosis regulator Bcl-2, BH" (*p *= 0.00296) for the APO class; "Transforming growth factor beta" (*p *< 0.00001), "Transforming growth factor beta (TGFb), N-terminal" (*p *< 0.00001), and "Growth factor, cystine knot" (*p *= 0.00002) for the GF class.

Obtained results demonstrate validity of the approach for the analysis of protein families, domains and related genes that we developed, implemented and made available within the *GFINDer *Web system.

## Discussion

Information about protein families and domains is paramount to understanding gene functional characteristics. As the most reliable source of such information we selected Pfam, the curated databank specifically concerning protein families and domains. In addition, we also considered InterPro databank, since it is the most comprehensive source of annotations on the subject. In fact, it contains data from many member databases such as UniProt, PROSITE, PRINTS, ProDom, SMART, TIGRFAMs, PIRSF, SUPERFAMILY, Gene3D, PANTHER, and also Pfam. Furthermore, InterPro provides parent/child relations among many of its entries. Such relations indicate hierarchical levels of detail between a parent entry and a more specific child entry. The *GFINDer Protein Families&Domains *modules we developed allow exploring and analyzing the available protein family and domain annotations by exploiting such relations. In the *Exploration *module, the user can select the level of detail at which exploring the protein family and domain annotations associated with a considered set of genes, or explore all levels at the same time (Figure 1). In the *Statistics *module, consecutive statistical tests are executed on each categorical annotation independently on its level of detail. Then, analysis results are shown listing each tested categorical annotation with its hierarchical level and the obtained *p*-value (Figures 2, 3 and 6). This simultaneously provides a comprehensive view of the statistical significance of all considered annotations and clearly highlights the evaluated protein characteristics with lowest *p*-value within each of the considered user-defined classes of genes, specifying also their granularity level. Validation results showed that this is correctly performed also when the same protein family or domain is associated with genes in different classes, as it happens for domains contained both in tyrosine kinase (TK) and tyrosine phosphatase (TP) proteins (Figure 1). In this case, although obtained *p*-values do not reach statistical significance, lower *p*-values properly indicate more relevant protein domains for each gene class (e.g. "Immunoglobulin subtype" (*p *= 0.21715) and "Immunoglobulin-like" (*p *= 0.234) for the TK class, or "Fibronectin, type III-like fold" (*p *= 0.16395) and "Fibronectin, type III" (*p *= 0.20151) for the TP class).

In the *Logistic Regression *module, the user can select the level of detail of the protein annotations to be included in the regression, or focus only on those independent annotations that are not linked by parent/child relations. Although logistic regression requires several samples (number of considered genes associated with each evaluated annotation) to provide reliable results [17], application results obtained with the new *GFINDer Logistic Regression *module are promising. They show that this module can support better interpretation of gene classes identified through gene expression experiments by finding the functional characteristics that better explain the considered gene classification. Furthermore, it can aid in better characterizing genes whose functional role is not yet completely understood, or even unknown, by estimating the probability of a gene to be functionally similar to a set of genes with certain functional characteristics. Thus, the implemented logistic regression analysis could contribute to unveil new biomedical knowledge about the considered genes.

Even if several tools are available for the analysis of gene annotations according to the Gene Ontology, at present to our knowledge only DAVID [18] and FatiGO+ [19] support analysis of protein family and domain controlled vocabularies, although in a more basic way than *GFINDer *does. Therefore, the novel implemented modules for evaluating protein family and domain annotations considerably enrich *GFINDer*, which is freely available on-line for non-profit use [7], and make it a unique valuable tool for genomic functional and phenotypic analysis of gene sets.

## Conclusion

The novel *GFINDer *modules for the evaluation of protein family and domain annotations of sets of genes can help in better interpreting high-throughput gene lists and in unveiling new functional knowledge about the considered genes, as our validation demonstrated. Thus, they can support a genomic approach in the understanding of fundamental biological processes and complex cellular mechanisms influenced by protein structural and functional characteristics related to domain composition of the codified proteins.

## Methods

### Structuring and hierarchical tree reconstruction of protein family and domain annotations

By using standard text-parsing procedures, we extracted protein accession numbers, Pfam IDs and protein family domain categories from the *uniprot sprot.dat *text file, which contains the whole protein information in the UniProt databank [20] and is freely available from the ExPASy FTP site [21]. Then, we structured the extracted controlled annotations in a *GFINDer *database table that we related to a previously created *gene2accession *correspondence table. This table contains nucleotide sequence and gene IDs, and the associated accession numbers of their protein products, which are freely available from the FTP site [22] of the Entrez Gene databank [23].

From the InterPro FTP site [24] we freely downloaded the MySQL dump of the entire InterPro databank [5]. Then, in *GFINDer *multi-database system we imported those InterPro tables containing controlled annotations of protein families, domains and functional sites and their hierarchical relations, and related them to the *gene2accession *correspondence table. Furthermore, we reconstructed the trees of parent/child hierarchical relations defined among the InterPro entries and structured them within the *GFINDer*/InterPro database in order to use them more efficiently during user protein annotation analysis.

### Implemented *GFINDer *system for protein family and domain investigation

*GFINDer *Web system is built as three-tier architecture based on a multi-database structure. In the first tier, the *data tier*, a MySQL DBMS manages all considered biomolecular annotations stored in different relational databases. In this tier, we added a specifically designed relational database where we stored and hierarchically structured all annotations retrieved about protein families and domains. To associate a protein family or domain with their codifying genes we considered the protein codes associated with a gene, as provided by the Entrez Gene database. Using Java programming language, we implemented procedures able to automatically import and keep updated, in *GFINDer data tier*, protein family and domain information and related gene annotations when new releases of them become available in UniProt, InterPro and Entrez Gene databanks. Particular procedures automatically reconstruct protein family and domain hierarchies according to the hierarchical information provided by InterPro, and structure them in the specific *GFINDer *database.

In *GFINDer processing tier *we used Microsoft ActiveX Data Object technology and Standard Query Language to interact with the MySQL DBMS server on the *data tier*, and created new management and analysis routines in Javascript and Active Server Page scripts. In the analysis routines categorical and logistic regression statistical approaches were implemented to evaluate protein family and domain category terms. They employ different tests (described in the *Statistical analysis *section below) to assess statistical significance of the over- and under-representation of categorical protein family and domain annotations in a group of user-classified genes.

In *GFINDer user tier*, which is composed of any client computer connected to the Web server on the *processing tier *through an Internet/intranet communication network, we employed Hyper Text Markup Language and Javascript to implement a Web graphic interface for the new developed *Protein Families & Domains *modules.

### Statistical analysis

Categorical statistical techniques were implemented in *GFINDer *to analyze protein family and domain controlled annotations. Because a gene may or may not be associated with a certain protein annotation category, the number of genes and their frequency, distribution, and probability of occurrence is calculated for each protein family and domain associated with the user-considered gene set. Several different statistical tests can be used to calculate a probability value of having *x *genes or fewer associated with a given annotation category. In *GFINDer *we implemented the *hypergeometric test *(more time-consuming), the *binomial test *(which is an asymptotic limit of the first for high number of genes), and the *exact Fisher test *(based on a two-way table crossing gene classes and protein family or domain categories) [6,25,26]. As usual in all significance tests, small *p*-values relate to relevant protein family or domain categories for a certain class of genes. However, depending on the number of considered genes and their associated protein families or domains, the number of performed statistical tests can be high. This can greatly increase the Type-I error associated with the tests, i.e. the probability of obtaining a significant *p*-value by chance when the null hypothesis is true (or the false-positive value, as it is known in the medical field). This requires corrections on the calculated *p*-values in order to obtain proper significances.

In *GFINDer *several correction methods for multiple tests are available. The simplest and strictest is the Bonferroni method that can be applied if the performed tests are independent [27]. It consists of changing the threshold *α *of each single test, from which every corresponding *p*-value is considered significant, in such a way that the Type-I error of the whole set of tests is maintained. The correction is the following: *α*_corrected _= *α */ N, where N is the number of performed tests. From a practical point of view this is equivalent to keep the usual threshold *α *for the performed tests and apply a correction to the observed *p*-values such that: *p*_corrected _= N * *p*. However, the Bonferroni method greatly reduces the power of detecting a specific hypothesis when the number of tests increases. False Discovery Rate (FDR) and Family-Wise Error-rate (FWE), an extension of Bonferroni method, are milder corrections and they are even suitable when independence among tests does not hold [28]. The former briefly consists in ordering the N *p*-values such that the maximum has rank N and the minimum has rank 1. Then, the correction to be applied is: *p*_corrected _= p * N / rank(*p*). The latter instead, in the implementation proposed by Benjamini and Hochberg [28], uses the following *p*-value correction: *p*_corrected _= p * (N - rank(*p*) + 1). All three methods above illustrated have been included in *GFINDer*. Among them the FDR, which is the mildest of the three and practically consists of defining the maximum acceptable number of obtained false-positive tests, is considered the most suitable correction method to be applied to genomic data.

### Logistic regression analysis

Logistic regression [29] is a variation of ordinary regression (y = b_0 _+ b_1_x_1 _+ b_2_x_2 _+ ... + b_i_x_i _+ ... + b_n_x_n_) that predicts the probability (*p*) of the occurrence of an outcome event (y) as a function of certain predictor variables (x_i_). It fits a special s-shaped curve by using the non linear function:

p=(ey1+ey)
 MathType@MTEF@5@5@+=feaafiart1ev1aaatCvAUfKttLearuWrP9MDH5MBPbIqV92AaeXatLxBI9gBaebbnrfifHhDYfgasaacH8akY=wiFfYdH8Gipec8Eeeu0xXdbba9frFj0=OqFfea0dXdd9vqai=hGuQ8kuc9pgc9s8qqaq=dirpe0xb9q8qiLsFr0=vr0=vr0dc8meaabaqaciaacaGaaeqabaqabeGadaaakeaacqWGWbaCcqGH9aqpdaqadaqaamaalaaabaGaeeyzau2aaWbaaSqabeaacqqG5bqEaaaakeaacqaIXaqmcqGHRaWkcqqGLbqzdaahaaWcbeqaaiabbMha5baaaaaakiaawIcacaGLPaaaaaa@3888@

which produces *p*-values between 0 (as y approaches minus infinity) and 1 (as y approaches plus infinity). In our implementation, we considered the following corresponding non linear equation, called logit(*p*):

logit(p)=ln⁡(p1−p)=y=b0+b1x1+b2x2+...+bixi+...+bnxn
 MathType@MTEF@5@5@+=feaafiart1ev1aaatCvAUfKttLearuWrP9MDH5MBPbIqV92AaeXatLxBI9gBaebbnrfifHhDYfgasaacH8akY=wiFfYdH8Gipec8Eeeu0xXdbba9frFj0=OqFfea0dXdd9vqai=hGuQ8kuc9pgc9s8qqaq=dirpe0xb9q8qiLsFr0=vr0=vr0dc8meaabaqaciaacaGaaeqabaqabeGadaaakeaacqqGSbaBcqqGVbWBcqqGNbWzcqqGPbqAcqqG0baDcqGGOaakcqWGWbaCcqGGPaqkcqGH9aqpcyGGSbaBcqGGUbGBdaqadaqaamaalaaabaGaemiCaahabaGaeGymaeJaeyOeI0IaemiCaahaaaGaayjkaiaawMcaaiabg2da9iabdMha5jabg2da9iabbkgaInaaBaaaleaacqaIWaamaeqaaOGaey4kaSIaeeOyai2aaSbaaSqaaiabigdaXaqabaGccqqG4baEdaWgaaWcbaGaeGymaedabeaakiabgUcaRiabbkgaInaaBaaaleaacqaIYaGmaeqaaOGaeeiEaG3aaSbaaSqaaiabikdaYaqabaGccqGHRaWkcqGGUaGlcqGGUaGlcqGGUaGlcqGHRaWkcqqGIbGydaWgaaWcbaGaeeyAaKgabeaakiabbIha4naaBaaaleaacqqGPbqAaeqaaOGaey4kaSIaeiOla4IaeiOla4IaeiOla4Iaey4kaSIaeeOyai2aaSbaaSqaaiabb6gaUbqabaGccqqG4baEdaWgaaWcbaGaeeOBa4gabeaaaaa@6740@

where *p *is the proportion of considered classified genes in the two evaluated classes; x_i _are the proportions of considered genes in the two evaluated classes that present the i characteristic; b_i _(with 1 ≤ i ≤ n) are the regression coefficients for the i characteristic; and b_0 _is the intercept, or constant term, of the regression. The absolute value of each b_i _coefficient indicates the importance of the corresponding i characteristic in contributing to the considered gene classification.

In order to solve the non linear equation, we used a straightforward Active Server Page and Javascript implementation of a standard iterative method [30] to minimize the log likelihood function. Such function is defined as the sum of the logarithms of the predicted probabilities of belonging to the first of the two evaluated classes for those considered genes indeed belonging to that class, and the logarithms of the predicted probabilities of belonging to the second of the two evaluated classes for those considered genes indeed belonging to that second class. Minimization is by Newton's method, with an elimination algorithm to invert and solve the simultaneous equations. No special convergence-acceleration techniques were used. The *null model *was used as starting guess for the iterations, i.e. all b_i _coefficients are zero and the b_0 _intercept is the logarithm of the ratio of the number of considered genes belonging to the first of the two evaluated classes to the number of considered genes belonging to the second class.

In addition to providing value, standard error and *p*-value of b_i _coefficients, our logistic regression implementation also produces an *odds ratio *associated with each predictor variable x_i _and its 95% confidence interval. The *odds *of an event is defined as the probability of the event occurring divided by the probability of the event not occurring. The *odds ratio *for a predictor variable x_i _denotes the relative amount by which the *odds *of belonging to the first of the two evaluated classes, for those considered genes that present the i characteristic, increases (*odds ratio *greater than 1.0) or decreases (*odds ratio *less than 1.0) when the value of the predictor variable x_i _is increased by 1.0 unit.

## Authors' contributions

MM was responsible for the overall conception and project coordination, was involved in design, development and testing of *GFINDer *software modules, and wrote this manuscript. EB developed *GFINDer *software module devoted to logistic regression, and was involved in their design and testing. AF developed the routines devoted to structuring and updating protein family and domain annotations within *GFINDer *database. FP provided supervision and funding of the project.
